# Supplementary material for: Principles of RNA processing from analysis of enhanced CLIP maps for 150 RNA binding proteins
Source: Genome Biol. 2020 Apr 6;21:90. doi: 10.1186/s13059-020-01982-9 (PMC7137325; doi:10.1186/s13059-020-01982-9)
Supplement: Supplementary file 3 — Additional file 3: Supplementary Figures S1-S8. [file 13059_2020_1982_MOESM3_ESM.docx]

**Supplementary Figures**

**
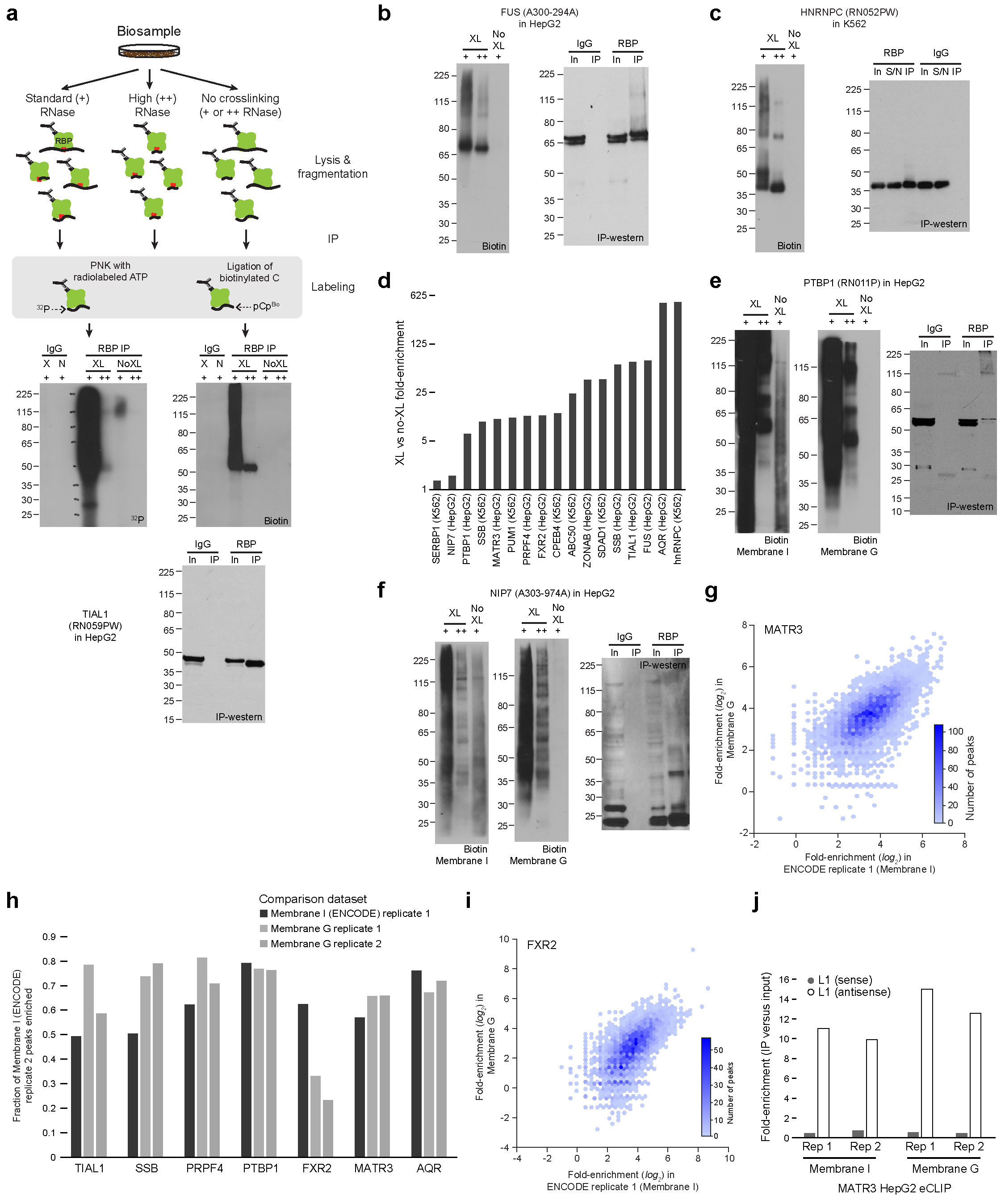
**

**Fig. S1. Visualization of RBP:RNA complexes with biotin-labeling.** (a) (top) Schematic of RBP:RNA visualization experiments, in which three samples are subjected to immunoprecipitation: crosslinked cells with standard (40U) RNase, crosslinked cells with high (333U) RNase, and non-crosslinked cells with either standard or high RNase. RNA was then labeled either through radiolabeling with T4 PNK and [γ-32P]-ATP followed by autoradiography, or with T4 RNA Ligase and pCp-Biotin followed by chemiluminescent imaging with streptavidin-conjugated horseradish peroxidase. (bottom) Example RNA imaging with ^32^P and biotin-labeling after TIAL1 immunoprecipitation, with standard IP-western shown below. (b-c) Biotin-based RNA labeling for (b) FUS in HepG2 and (c) HNRNPC in K562. (d) Bars indicate the fold-enrichment in biotin-labeled RNA signal between crosslinked versus non-crosslinked samples (with 40U RNase) for the size range from which RNA is isolated (from protein to 75 kDa above). Shown is data from membrane I. Quantification was performed in ImageJ. (e-f) Biotin-based RNA labeling for (e) PTBP1 in HepG2 and (f) NIP7 in HepG2. For each, immunoprecipitated sample was labeled with pCp-Biotin and split in half, with one half transferred to nitrocellulose membrane from supplier I, and the other half transferred to nitrocellulose membrane from supplier G. (right) IP-western experiment from the paired eCLIP experiments). (g) Density plot indicates the number of eCLIP peaks for MATR3 in HepG2 identified as significant in ENCODE replicate 2 that have the indicated fold-enrichment in (x-axis) ENCODE replicate 1 (performed with membrane I) versus (y-axis) a new eCLIP replicate performed with membrane G. Color indicates the number of points within each hexagon. (h) Bars indicate the fraction of significantly-enriched peaks in ENCODE replicate 2 (performed with membrane I) that are also significantly enriched in (black) ENCODE replicate 1 (membrane I) or (gray) replicates 1 or 2 of a new eCLIP experiment performed with membrane G in the same cell type with the same antibody. (i) Density plot indicates the number of eCLIP peaks for FXR2 in HepG2 identified as significant in ENCODE replicate 2 that have the indicated fold-enrichment in (x-axis) ENCODE replicate 1 (performed with membrane I) versus (y-axis) a new eCLIP replicate performed with membrane G. Color indicates the number of points within each hexagon. (j) Bars indicate the fold-enrichment for read density at (white) sense or (gray) antisense L1 elements in MATR3 eCLIP in HepG2.

**
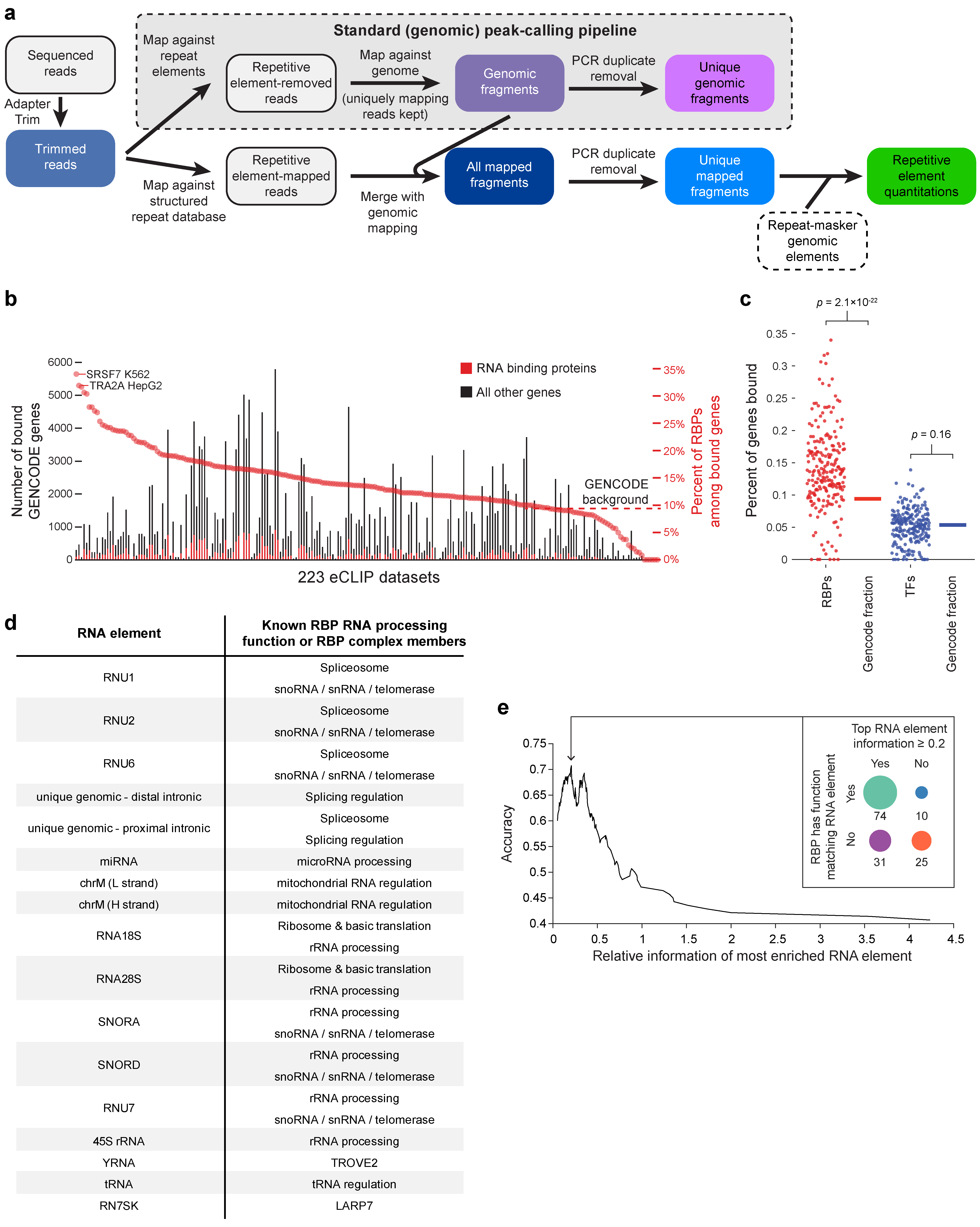
**

**Fig. S2. Quantification of genomic signal versus repetitive elements and other non-uniquely mapped reads.** (a) Schematic indicates major steps for (grey box) the standard eCLIP peak calling pipeline (which uses uniquely mapping reads only) and the repetitive element quantification pipeline. (b) Stacked bars indicate number of (red) RBPs and (black) all other GENCODE genes with eCLIP peaks in each dataset. Red circles indicate the percent of bound genes that are RBPs. eCLIP datasets are sorted by percent of RBPs. (c) Percent of genes that are (red) RBPs or (blue) transcription factors (TFs) are indicated for all 223 eCLIP datasets, compared with the fraction of RBPs out of all GENCODE genes that contain at least one peak in any of the 223 eCLIP datasets. Significance was determined by two-sided Kolmogorov-Smirnov test. (d) Table indicates RNA elements paired with canonical functional annotations for their respective ribonucleoprotein complexes, or (for YRNA and RN7SK) well-characterized RBP members of those respective ribonucleoprotein complexes. (e) Graph indicates accuracy (defined as (TP + TN) / (TP + TN + FP + FN)) for the top element (by relative information) from eCLIP for an RBP matching an annotated function for that RBP (see Methods). Analysis was performed on 140 eCLIP datasets with functional annotation matching those listed in (d).

**
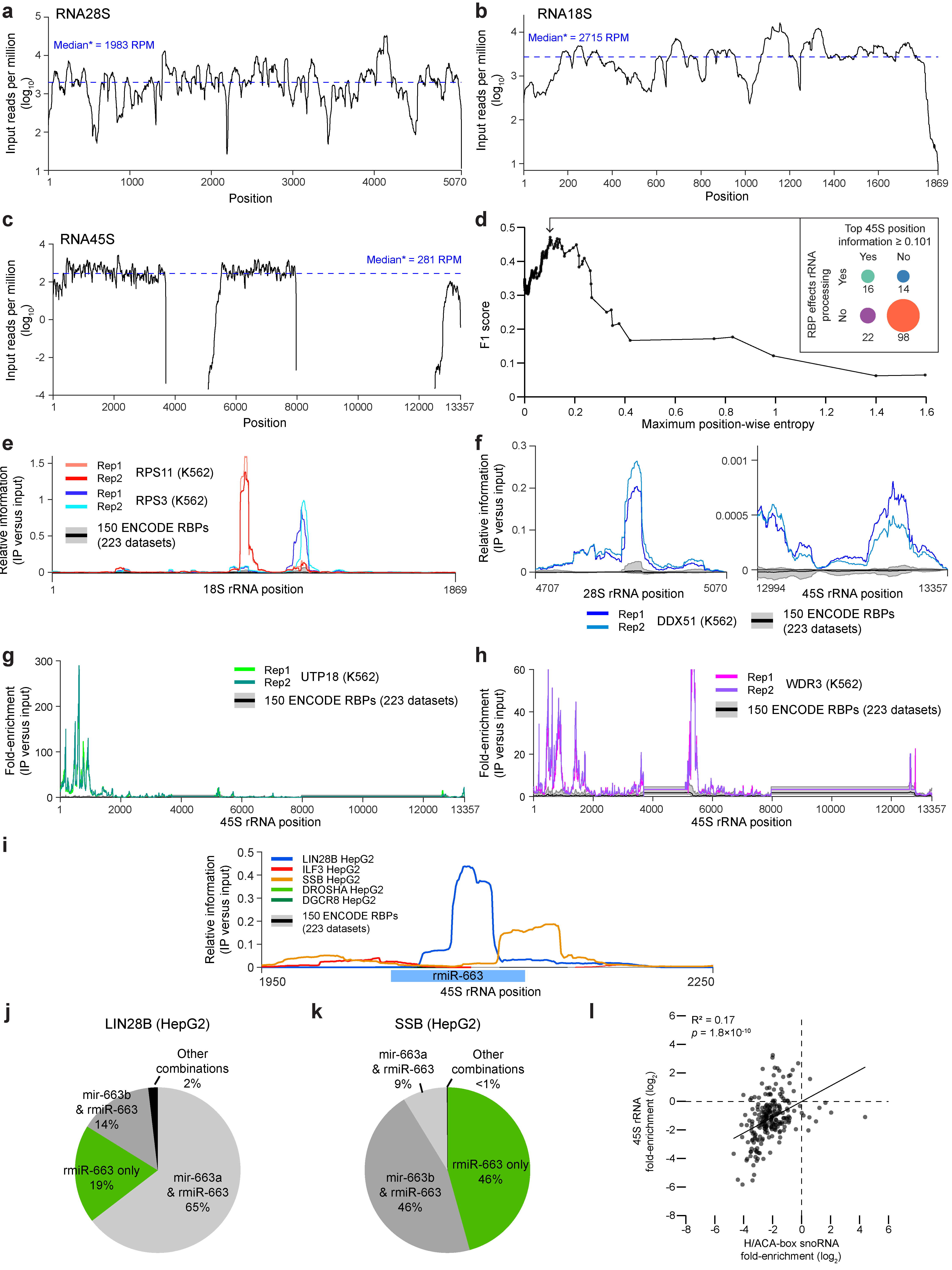
**

**Fig. S3. eCLIP enrichment for rRNA links RBPs with ribosomal RNA processing.** (a-c) Line indicates average reads (per million non-PCR duplicate reads) mapping to each position across (a) 28S rRNA, (b) 18S rRNA, and (c) 45S rRNA. Blue line indicates median (calculated across all non-zero positions). Due to inability to distinguish between reads mapping to the 18S (or 28S) mature rRNA versus identical regions in the 45S precursor, reads mapping equally well to both are assigned to the 18S (or 28S), leading to gaps in the 45S plot. (d) Plot indicates (y-axis) F1 score for (x-axis) indicated relative information cutoffs, using RBPs with rRNA processing defects observed upon RNAi knockdown in Tafforeau *et al*. [[28](#_ENREF_28)] as the ‘true positive’ reference set. (e-i) Lines indicate (y-axis) either fold-enrichment or relative information as indicated for (e) RPS11 in K562 and RPS3 in K562 for 18S rRNA, (f) DDX51 in K562 for 3’ end regions within (left) 28S rRNA and (right) 45S rRNA, (g) UTP18 in K562 for the 45S rRNA, (h) WDR3 in K562 for the 45S rRNA, and (i) indicated RBPs for the indicated 45S rRNA region flanking a putative ribosomal RNA-encoded microRNA (rmiR-663). For each, black line indicates mean and grey region indicates 10^th^ to 90^th^ percentile across all 223 eCLIP datasets. (j-k) Pie chart indicates for (j) LIN28B eCLIP in HepG2 or (k) SSB in HepG2 the fraction of reads that either map (green) with fewer mismatches to the rmiR-663 or (grey) to various combinations of rmiR-663 and genomic-encoded miR-663a/b and related family members (as shown in Fig. 3f). (l) Points indicate fold-enrichment in each eCLIP dataset for (x-axis) H/ACA-box snoRNAs versus (y-axis) the 45S rRNA precursor. Pearson correlation and significance was calculated in MATLAB.

**
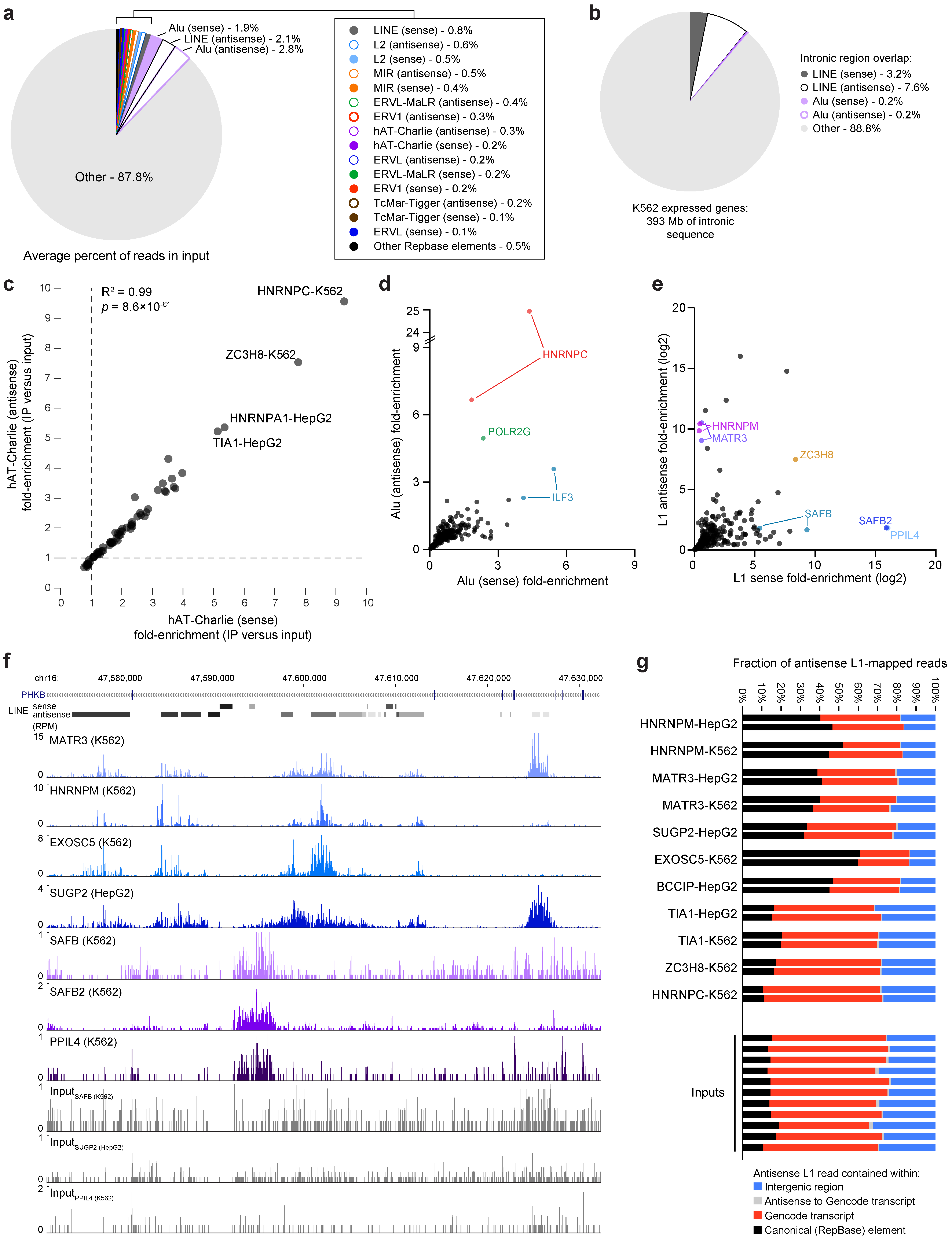
**

**Fig. S4. RBP association at retrotransposable and other repetitive elements.** (a) Pie chart indicates the percent of reads (averaged across 223 eCLIP inputs) that map either uniquely to the genome within a RepeatMasker-predicted element, or are assigned to the consensus element sequencing in the family-aware mapping approach. Shown are RepBase element classes with average read density of at least 0.1%. Filled circles indicate sense elements, with open circles indicating antisense elements. (b) Pie chart indicates the fraction of intronic bases that overlap RepeatMasker-predicted L1 and Alu (sense and antisense) elements. Shown are genes with TPM≥1 in K562 cells, using the transcript isoform with the highest abundance in K562 rRNA-depleted RNA-seq. (c-e) Points indicate fold-enrichment (averaged across two biological replicates) for 223 eCLIP datasets for (c) hAT-Charlie, (d) Alu, and (e) L1 elements. In each, x-axis indicates sense and y-axis indicates antisense elements. Pearson R^2^ and significance was determined in MATLAB. (f) Genome browser image indicates read density for L1 sense- and antisense-enriched RBPs in intronic regions in *PKHB.* (g) Stacked bars indicate the fraction of antisense L1-assigned reads that were derived from mapping to (black) the canonical RepBase L1 element, (red) antisense L1 elements within Gencode v19 transcripts, (grey) to antisense L1 elements that are on the opposite strand from a Gencode v19 transcript, or (blue) antisense L1 elements in intergenic regions.


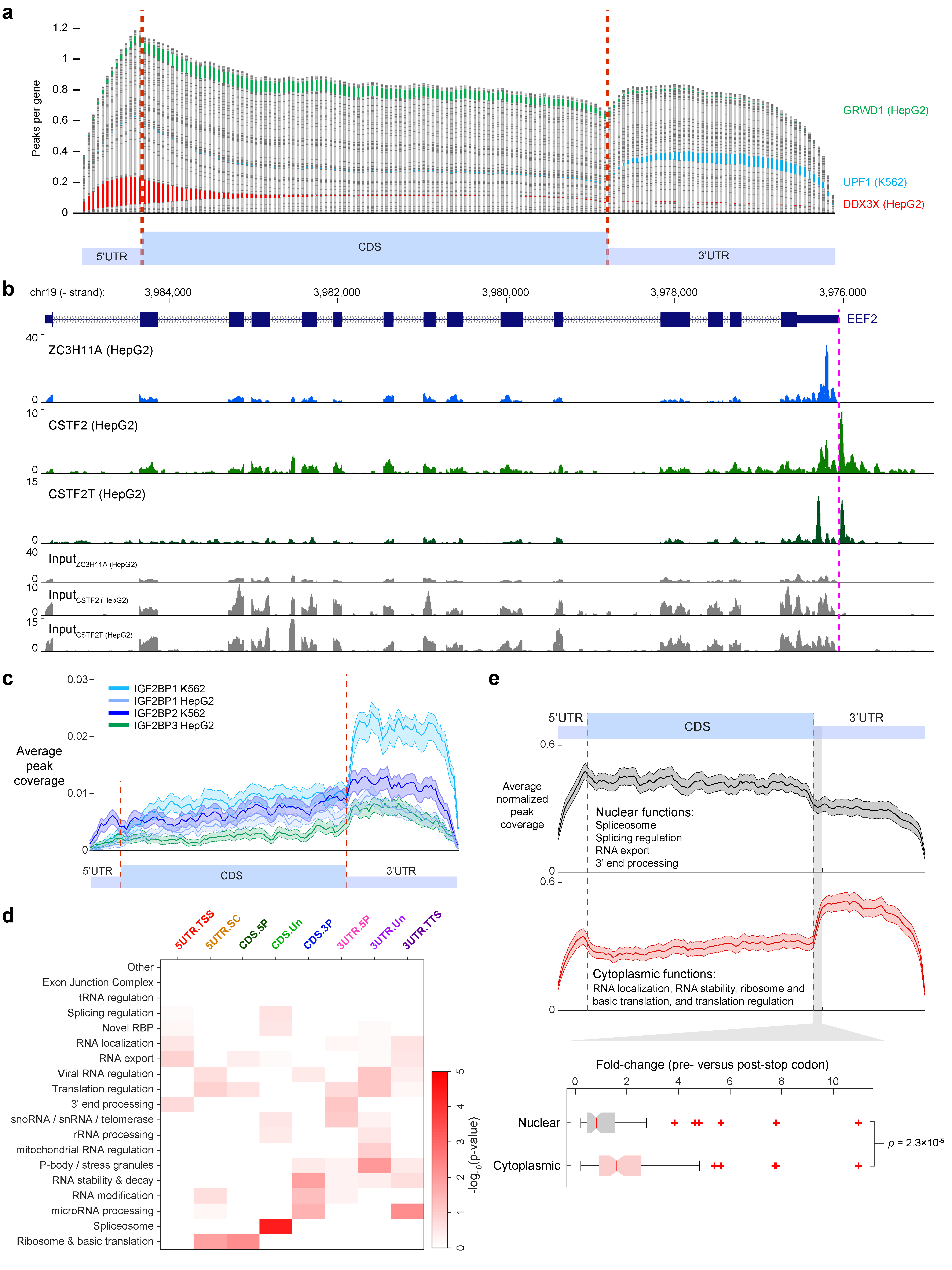


**Fig. S5. mRNA metagene profiles from eCLIP correspond to RBP RNA processing roles.** (a) To create meta-mRNAs, each gene was normalized to 13 5’UTR, 100 CDS, and 49 3’UTR bins (based on average lengths among expressed transcripts in K562 cells). Stacked bars indicate the average number of significant and reproducible peaks for each RBP in each bin. (b) Tracks show read density in ZC3H11A, CSTF2, and CSTF2T eCLIP for *EEF2*. CSTF2 and CSTF2T show continuing read density past the annotated polyadenylation site. (c) Lines indicate average number of significant reproducible peaks per gene for bins across a meta-mRNA for IGF2BP family eCLIP. Shaded region indicates 5^th^ and 95^th^ percentile from 100 bootstrap samples. (d) Heatmap indicates significance (by Fisher’s Exact test, or Yates’ Chi-Square test where appropriate) of overlap between eCLIP datasets in indicated meta-mRNA cluster (x-axis) versus annotated RBP functions (y-axis). (e) (top) Lines indicate average normalized peak coverage for RBPs annotated with nuclear (*n* = 76) and cytoplasmic (*n* = 89) functions as indicated, with some RBPs present in both lists. Shaded region indicates standard error of the mean. (bottom) Box indicates 25^th^ to 75^th^ percentile (with median in red) of ratio between normalized peak coverage at bin 117 (post-stop codon) versus 113 (pre-stop codon). Significance was determined by two-sided Kolmogorov-Smirnov test.


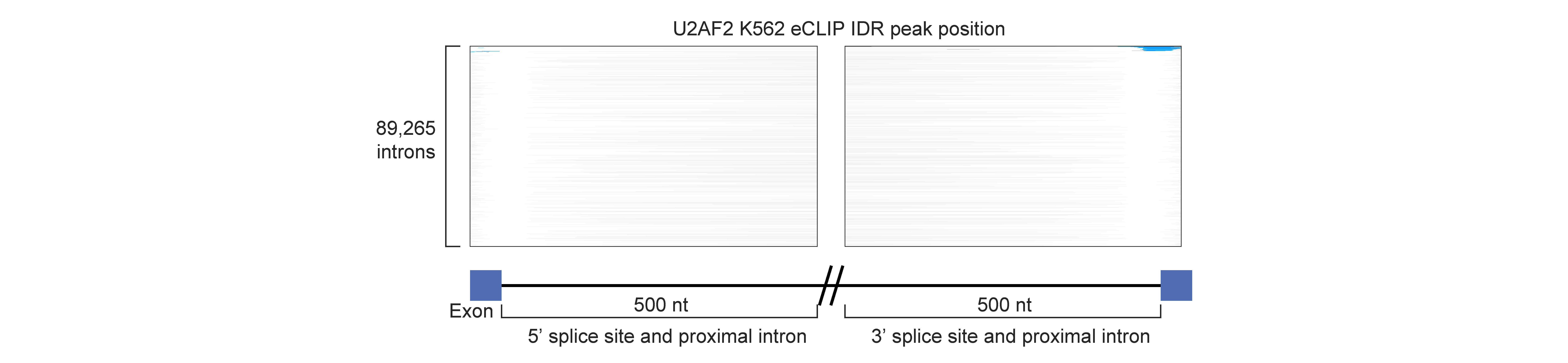


**Fig. S6. Meta-exon plots reveal intronic regulatory roles.** Each line indicates the presence (in blue) of a reproducible U2AF2 K562 eCLIP peak for all 89,265 introns identified within genes with TPM ≥ 1 in K562 total RNA-seq. Region shown includes 500 nt of proximal intron and 50nt of exon flanking the 5’ and 3’ splice sites.

**
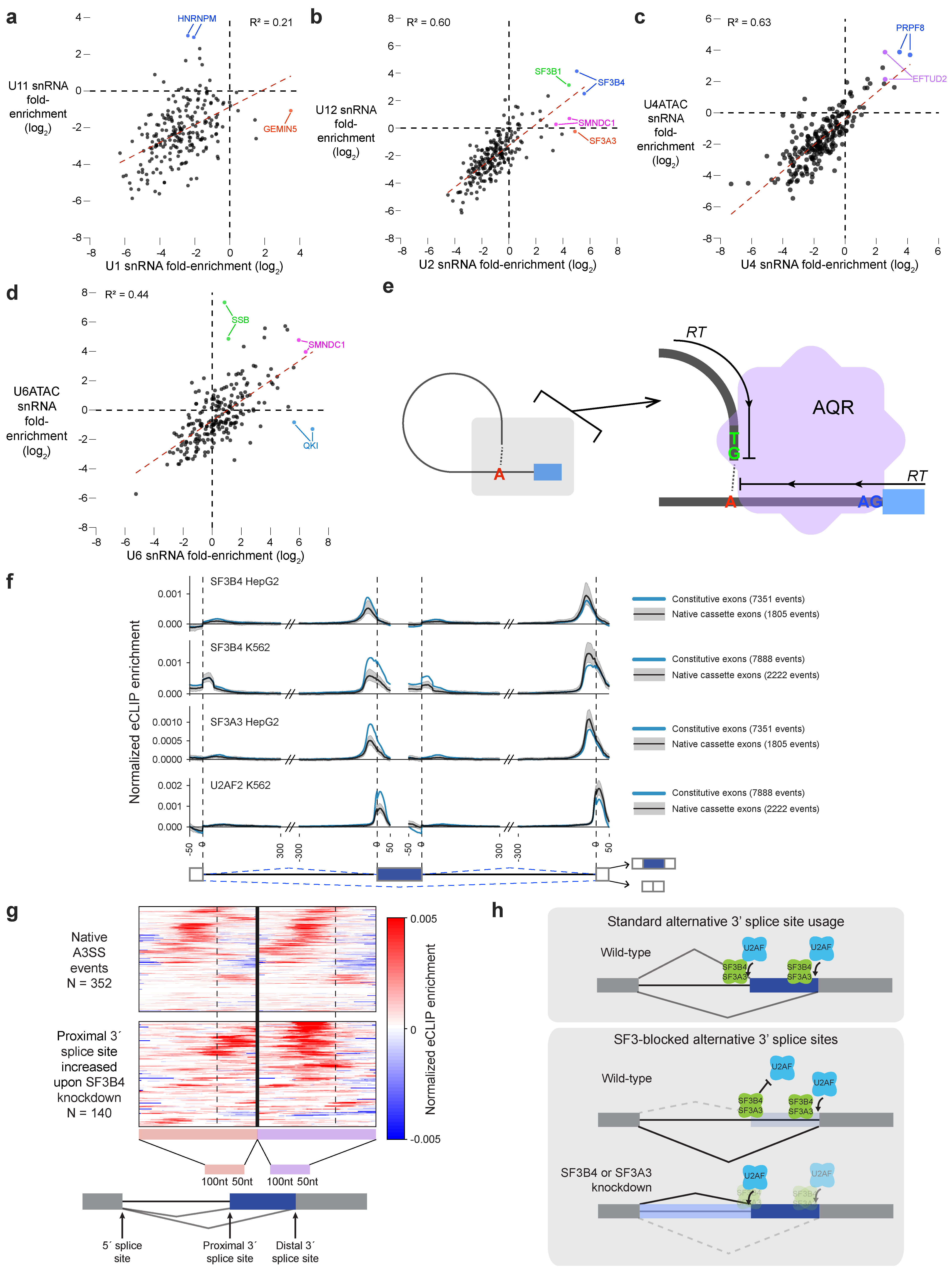
**

**Fig. S7. Insights from eCLIP of splicesome-associated RBPs.** (a-d) Points indicate fold-enrichment in 223 eCLIP datasets for indicated (x-axis) major U2 spliceosomal snRNAs versus (y-axis) orthologous snRNAs in the U11/U12 minor spliceosome. (e) Model for AQR association with intronic lariats. Reverse transcription terminates at the lariat, generating reads with 5’ ends at the 5’ splice site as well as the position following the branch point adenosine. (f) Normalized splicing maps of SF3B4, SF3A3, and U2AF2 for (blue) constitutive exons versus (black) a set of 'native' cassette exons (nSE) with 0.05 < inclusion rate < 0.95 in controls. Lines indicate average eCLIP read density in IP versus input for indicated exon categories. Shaded area indicates 0.5th and 99.5th percentiles observed from 1000 random samplings of native events. The displayed region shown extends 50 nt into exons and 300 nt into introns. (g) Heatmap indicates normalized eCLIP signal for SF3B4 in HepG2 cells at alternative 3’ splice site events either (top) alternatively spliced in wild-type cells or (bottom) events with increased usage of the extended 3’ splice site upon SF3B4 knockdown. The region shown extends 50 nt into exons and 100 nt into introns. (h) Model for SF3B4 and SF3A3 blockage of 3’ splice site recognition by U2AF. At SF3-blocked alternative 3’ splice site events, knockdown of SF3 components leads to either usage of the upstream (proximal) 3’ splice site, or retention of the intron.


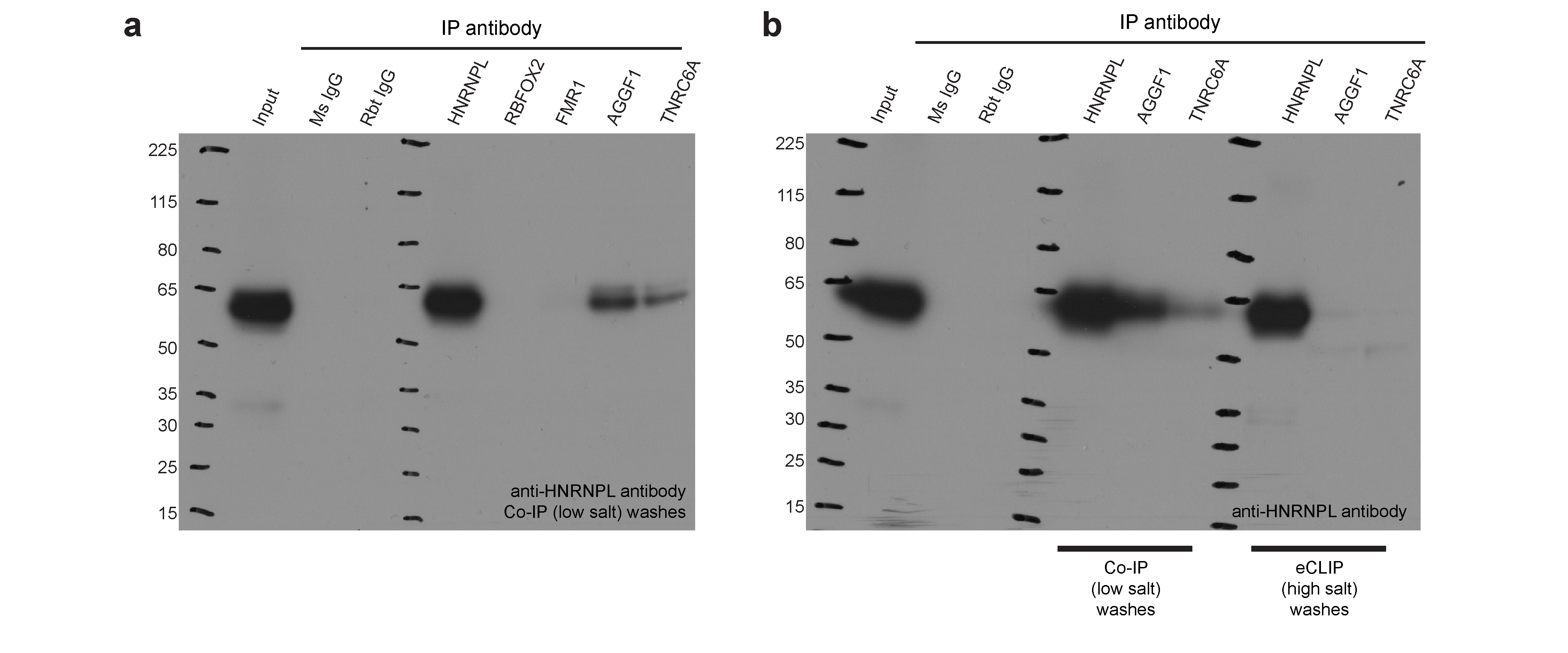


**Fig. S8. RBP co-association predicts known and novel RNP complexes.** (a) Western blot for HNRNPL performed using 5 RBPs for immunoprecipitation (HNRNPL, RBFOX2, FMR1, AGGF1, and TNRC6A). Immunoprecipitation was performed using low-salt washes only. Also shown are immunoprecipitation using IgG isotype control for mouse and rabbit. (b) Western blot for HNRNPL using 3 RBPs for immunoprecipitation (HNRNPL, AGGF1, and TNRC6A), performed in two conditions: high salt washes (using standard eCLIP wash buffers), and low salt washes.
