# Supplementary material for: Principles of RNA processing from analysis of enhanced CLIP maps for 150 RNA binding proteins
Source: Genome Biol. 2020 Apr 6;21:90. doi: 10.1186/s13059-020-01982-9 (PMC7137325; doi:10.1186/s13059-020-01982-9)
Supplement: Supplementary file 6 — Additional file 6: Review history. [file 13059_2020_1982_MOESM6_ESM.docx]

Review History

**First round of review**

**Reviewer 1**

**Are you able to assess all statistics in the manuscript, including the appropriateness of statistical tests used?** No, I do not feel adequately qualified to assess the statistics.

**Comments to author:**

This manuscript by Van Nostrand and colleagues is based on an impressive body of eCLIP data available through ENCODE. Several other publications have already used this data, including by the authors themselves, but the work here is particularly focussed on aspects of RNA processing and making best use of reads that mapped to repetitive elements in a family-aware manner. The manuscript establishes a clear rationale for the work and conclusions are generally supported by the data analyses. It is also written quite clearly, allowing the reader to follow broadly, what data transformations were done etc. Nevertheless, I suggest that the authors carefully assess one more time whether they have provided all necessary explanations of their data transformations, definitions of acronyms and abbreviations and how figure legends are used to provide more technical information to the reader. I found a few problems in the methods, for example, equations are presented that use terms like MN, MX, TP, TN, FP etc, which are not defined. Given that this affects how the somewhat less than common parameter 'accuracy' is used, I think it should be properly explained.

The family-aware mapping to multi-copy elements is well explained in the methods, but I think it would be nice to have a graphic representation of this approach as a schematic in one of the main figures.

Clearly, the manuscript advances the field, however, I would have appreciated a clear statement as to how the present work is distinct of papers by the same authors that have used the same data. For example, reference 20 is a 2017 submission to BioRkiv, which appear not to have been published elsewhere as yet. What is the current state with publishing the 2017 study? Is it to be viewed as a companion paper to the present work? If so, how exactly are they thematically distinct?

**Reviewer 2**

**Are you able to assess all statistics in the manuscript, including the appropriateness of statistical tests used?** Yes, and I have assessed the statistics in my report.

**Comments to author:**

In this manuscript Van Nostrand et al describe the protein-RNA interaction landscape for hundreds of RNA binding proteins. This integrative analysis of binding profiles reveals common themes and functional relationships between RBPs as well as discovering potential new roles for RBPs in rRNA processing and pre-mRNA splicing (Lin28B and AQR, respectively). The other important contribution this analysis makes is in revealing the widespread associations of RBPs with RNA transcripts derived from repetitive elements. This systematic analysis of repeat-derived binding sites raises the intriguing hypothesis that RBPs function in retrotransposition and that repeat-derived sequences may function in post-transcriptional gene regulation. The family-aware mapping approaches described here should enable other labs to investigate the functional relevance of RBP-repeat RNA interactions in a wide array of model systems. Overall the statistical analyses are well justified and appropriate.

The primary concerns with the manuscript focus on presentation and clarity. The main figures are information-rich and should be included in the final paper. However, there are aspects of the manuscript that distract from the papers message. Most notable are the technical aspects of alternative end labeling approaches and membrane selection. It does not appear that any of the data generated in the study used the biotin pCp labeling system (I could be wrong). I think this manuscript would be stronger if this information was omitted, or perhaps included in a supplemental methods section.

Another way in which the presentation could be improved is by moving from expected results to unexpected findings. It makes sense to me to present Figure 1 first, then move through the results related to fairly well understood classes of RNA transcripts such as rRNA and pre-mRNA. Then having established in the readers mind the strong connection to what is known/expected, the repeat analysis (Figures 2 and 4, plus supplements) could be presented. Figure 8 remains an excellent conclusion to the manuscript.

Overall, this is an important and impressive study, but the manuscript could benefit from a curatorial approach prior to publication. The issues described above do not diminish from my enthusiasm for this work.

**Authors Response**

**Point-by-point responses to the reviewers’ comments:**

Reviewer #1: This manuscript by Van Nostrand and colleagues is based on an impressive body of eCLIP data available through ENCODE. Several other publications have already used this data, including by the authors themselves, but the work here is particularly focussed on aspects of RNA processing and making best use of reads that mapped to repetitive elements in a family-aware manner. The manuscript establishes a clear rationale for the work and conclusions are generally supported by the data analyses. It is also written quite clearly, allowing the reader to follow broadly, what data transformations were done etc.

We appreciate the reviewer’s positive comments.

Nevertheless, I suggest that the authors carefully assess one more time whether they have provided all necessary explanations of their data transformations, definitions of acronyms and abbreviations and how figure legends are used to provide more technical information to the reader. I found a few problems in the methods, for example, equations are presented that use terms like MN, MX, TP, TN, FP etc, which are not defined. Given that this affects how the somewhat less than common parameter 'accuracy' is used, I think it should be properly explained.

We have expanded explanations of our data transformations, acronym definitions and abbreviations to the Methods as suggested.

The family-aware mapping to multi-copy elements is well explained in the methods, but I think it would be nice to have a graphic representation of this approach as a schematic in one of the main figures.

We have added a new Figure 2a as suggested here, with the old Figure 2a (a more computational-focused schematic) moved to Supplemental Fig. 2a.

Clearly, the manuscript advances the field, however, I would have appreciated a clear statement as to how the present work is distinct of papers by the same authors that have used the same data. For example, reference 20 is a 2017 submission to BioRkiv, which appear not to have been published elsewhere as yet. What is the current state with publishing the 2017 study? Is it to be viewed as a companion paper to the present work? If so, how exactly are they thematically distinct?

We have clarified the description of the links to that manuscript in the revision. To clarify here, we view this manuscript as a companion paper to reference 20, which is currently in press at *Nature*. The *Nature* article focuses on two major themes: (1) a description of the primary ENCODE RBP data resource and database reference for the eCLIP, knockdown RNA-seq, RNA Bind-N-Seq, ChIP-seq, and RBP immunofluorescence / localization data, and (2) utilization of that resource in integrative analyses combining multiple data-types (for example, generation of splicing regulatory maps by integrating eCLIP with knockdown/RNA-seq for the same RBP). This manuscript delves far deeper into the eCLIP resource itself and addresses questions we were not able to in the *Nature* manuscript (due to length constraints and scope).

Reviewer #2:

In this manuscript Van Nostrand et al describe the protein-RNA interaction landscape for hundreds of RNA binding proteins. This integrative analysis of binding profiles reveals common themes and functional relationships between RBPs as well as discovering potential new roles for RBPs in rRNA processing and pre-mRNA splicing (Lin28B and AQR, respectively). The other important contribution this analysis makes is in revealing the widespread associations of RBPs with RNA transcripts derived from repetitive elements. This systematic analysis of repeat-derived binding sites raises the intriguing hypothesis that RBPs function in retrotransposition and that repeat-derived sequences may function in post-transcriptional gene regulation. The family-aware mapping approaches described here should enable other labs to investigate the functional relevance of RBP-repeat RNA interactions in a wide array of model systems. Overall the statistical analyses are well justified and appropriate.

We thank the reviewer for his/her appreciation of the work described here.

The primary concerns with the manuscript focus on presentation and clarity. The main figures are information-rich and should be included in the final paper. However, there are aspects of the manuscript that distract from the papers message. Most notable are the technical aspects of alternative end labeling approaches and membrane selection. It does not appear that any of the data generated in the study used the biotin pCp labeling system (I could be wrong). I think this manuscript would be stronger if this information was omitted, or perhaps included in a supplemental methods section.

We appreciate the reviewer’s concern with the fit of this section. The reviewer is correct that the pCp labeling method was developed (and thus, the potential issue with nitrocellulose membrane suppliers was identified) after the data described here was generated; however, we included it here because we feel that this is an important caveat to include as part of this work (instead of as an additional manuscript, where it may be missed by researchers utilizing the eCLIP data resource). We will discuss with the Editor as to whether the biotin pCp labeling is better suited as supplemental methods.

Another way in which the presentation could be improved is by moving from expected results to unexpected findings. It makes sense to me to present Figure 1 first, then move through the results related to fairly well understood classes of RNA transcripts such as rRNA and pre-mRNA. Then having established in the readers mind the strong connection to what is known/expected, the repeat analysis (Figures 2 and 4, plus supplements) could be presented. Figure 8 remains an excellent conclusion to the manuscript.

We appreciate the reviewer’s suggestion. We have tried multiple approaches to the layout of this manuscript; the challenge we found with layouts similar to what the reviewer is suggesting is that we found it hard to describe mapping to rRNA and snRNAs without first describing the repeat mapping approach (and giving the overall context in Fig 2 / Sup. Fig 2).

Overall, this is an important and impressive study, but the manuscript could benefit from a curatorial approach prior to publication. The issues described above do not diminish from my enthusiasm for this work.
